# Development of Porous Piezoceramics for Medical and Sensor Applications

**DOI:** 10.3390/ma8125498

**Published:** 2015-12-21

**Authors:** Erling Ringgaard, Frans Lautzenhiser, Louise M. Bierregaard, Tomasz Zawada, Eric Molz

**Affiliations:** 1 Meggitt Sensing Systems Denmark, Porthusvej 4, Kvistgaard DK-3490, Denmark; louise.bierregaard@meggitt.com (L.M.B.); tomasz.zawada@meggitt.com (T.Z.); 2 Meggitt Sensing Systems Indiana, 8431 Georgetown Road, Indianapolis, IN 46268, USA; frans.lautzenhiser@meggitt.com (F.L.); eric.molz@meggitt.com (E.M.)

**Keywords:** piezoelectricity, ultrasound, porous ceramics, acoustic impedance

## Abstract

The use of porosity to modify the functional properties of piezoelectric ceramics is well known in the scientific literature as well as by the industry, and porous ceramic can be seen as a 2-phase composite. In the present work, examples are given of applications where controlled porosity is exploited in order to optimise the dielectric, piezoelectric and acoustic properties of the piezoceramics. For the optimisation efforts it is important to note that the thickness coupling coefficient *k*_t_ will be maximised for some non-zero value of the porosity that could be above 20%. On the other hand, with a good approximation, the acoustic velocity decreases linearly with increasing porosity, which is obviously also the case for the density. Consequently, the acoustic impedance shows a rather strong decrease with porosity, and in practice a reduction of more than 50% may be obtained for an engineered porous ceramic. The significance of the acoustic impedance is associated with the transmission of acoustic signals through the interface between the piezoceramic and some medium of propagation, but when the porous ceramic is used as a substrate for a piezoceramic thick film, the attenuation may be equally important. In the case of open porosity it is possible to introduce a liquid into the pores, and examples of modifying the properties in this way are given.

## 1. Introduction

In many cases, functional materials are selected as a trade-off between a set of requirements. When these requirements are in contradiction, a composite could be a more optimal material, combining at least two materials with very different properties. The preferred structure of a composite depends on the physics governing the application as well as on manufacturability.

Porous materials can be considered composites where the secondary phase is air. Many examples are known from other fields, e.g., fur, wool *etc*. for thermal insulation, foam for mechanical damping, and biological bone structure for optimizing strength/mass. As will be apparent from this study, piezoelectric ceramics is a field where a number of significant advantages are obtained by introducing porosity. Before proceeding with a brief literature overview for porous piezoceramics, the terminology of composite electroceramics will be reviewed.

In a classical paper, Newnham *et al*. introduced a very practical notation for composites based on the concept of connectivity [1]. The connectivity of a phase is the number of dimensions in which it is fully connected and thus can take the values of 0, 1, 2 and 3. Furthermore, by convention, the active (e.g., piezoelectric) phase is mentioned first in the case of a diphasic composite. Some common examples of the use of Newnham’s notation are 2-2 composites for multilayer structures, 0-3 for piezoceramic particles embedded in a polymer matrix, and 3-0 or 3-3 for a porous piezoceramic with closed or open porosity, respectively.

Banno used a unit-cell model based on modified cubes to make calculations of the properties of 1-3, 0-3 and 3-0 composites and gave porous ceramics as an example of the 3-0 case [2]. A pioneering study of porous piezoceramics was performed in 1986 by Wersing *et al*. [3], including a thorough introduction to the theory of these materials and some detailed measurements. The group of Galassi has explored a number of preparation methods and their influence on piezoelectric properties; see, for example, [4,5]. Important examples of methods for preparing porous ceramics include replica techniques (e.g., sponges for 3-3 composites), sacrificial template methods (either synthetic such as PMMA or natural such as wax), and direct foaming methods [5]. It should be noted that a certain degree of porosity can be obtained by simply reducing the sintering temperature with respect to the dense ceramic, but if high porosity is needed, one of the aforementioned methods are preferred. Bowen and co-workers have investigated porous piezoceramics for sensor applications where the high hydrostatic figure of merit is very attractive, both experimentally and by modelling [6,7,8]. Rybyanets has performed extensive work on porous piezoceramics, including preparation methods, theory, modelling and functional characterisation, *cf*. the review article [9]. Reference [10] described a study of pyroelectric and complex piezoelectric properties as a function of porosity. A study of a commercial, porosity-engineered piezoceramic material, Ferroperm™ Pz37 (one of the subjects of the present work), with special emphasis on frequencies in the megahertz range was published in 2004 [11].

Some of the first work on porous piezoceramic thick films was published by Kosec *et al*. in 2001 [12]. Since then various aspects of high-frequency applications have been explored [13], and it is now also a commercial product (see e.g., [14]). 

The purpose of this paper is to show two rather different cases of porous piezoceramics with important commercial applications. In the first case, piezoceramic discs have been prepared using a conventional bulk ceramic process involving uniaxial pressing, and a consistent level of porosity has been obtained by adding an organic pore former in the powder stage. The intended application for these discs is so-called downhole transducers to be used in the oil industry for acoustic pulse-echo measurements in logging-while-drilling tools. The operating conditions at the bottom of an oil well are quite severe and especially the pressures sometimes exceeding 200 MPa are a challenge. A common way of dealing with the high external pressure is to use a transducer design where the interior of the transducer is filled with a fluid maintained at the same pressure. The initial part of the work performed here therefore consists in characterising the porous piezoceramic before and after impregnation with a suitable fluid in order to evaluate the change in key properties. Next, a simple transducer has been manufactured using the porous ceramic and pulse-echo measurements have been performed.

**Figure 1:**
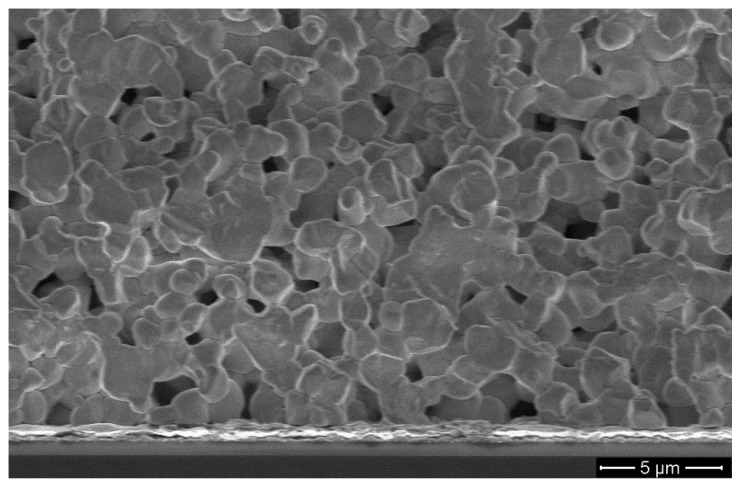
SEM image of typical microstructure of the piezoceramic thick film, clearly showing the high porosity [16]. © 2015 IEEE. Reprinted, with permission, from doi:10.1109/ULTSYM.2015.0277.

The second case of porous piezoceramics is PZT thick films that are deposited on a substrate by screen printing. The porosity of the thick films is a consequence of a reduced sintering temperature compared to the bulk case and the shrinkage being limited by adhesion to the substrate. An example of such a porous thick film is shown in Figure 1. The thickness and characteristic properties of these films make them suited for ultrasonic imaging at frequencies in the range 10 MHz to 30 MHz, both single-element transducers and multi-element arrays. When the former type is used for ultrasonic imaging, it needs to be moved (scanned) mechanically in order to produce a 2-dimensional image [14]. However, one of the advantages of multi-element array transducers is that electronic beam steering can be performed, as well as electronic beam focusing [15]. The present work includes examples of the design and performance of both single-element and multi-element transducers in pulse-echo measurements.

## 2. Results and Discussion

### 2.1. Pulse-Echo Transducers for Downhole Measurements

#### 2.1.1. Characterisation of Bulk Discs

Porous piezoceramic bulk samples of the material Ferroperm™ Pz37 [17] were prepared as described in the experimental section and a number of relevant properties were measured. To begin with, the density *ρ* is determined from the mass and geometrical dimensions. The relative permittivity in the 33 direction (*ε*_33,r_) is determined from the capacitance measured at 1 kHz and the geometric dimensions. The piezoelectric charge coefficient *d*_33_ has been measured directly, and the voltage coefficient has then been calculated from the following expression:

(1) $$g_{33}=\frac{d_{33}}{\varepsilon_{33,\text{r}}\varepsilon_{0}}$$

The frequency constants for the same modes (*N*_t_ and *N*_p_, respectively) have been determined from the relevant resonance frequency as shown in the following example for the thickness mode (where *h* is the thickness):

(2) $$N_{\text{t}}=f_{\text{r,t}}h$$

The coupling coefficients in the thickness and the planar mode (*k*_t_ and *k*_p_, respectively) have been determined according to common equations given in reference [18] on the basis of impedance spectra. As described by Alemany *et al*. [19], lossy piezoelectric materials (such as porous piezoceramics) offer special challenges for characterisation using impedance spectra and a detailed study would involve determining dielectric, piezoelectric and mechanical properties as complex quantities, *cf*. [10]. However, this study is focused on properties with direct importance for pulse-echo applications so only mechanical losses are considered.

The mechanical quality factor *Q*_m_ has a special significance for pulse-echo applications, since a high *Q*_m_ (*i.e.*, low mechanical loss) leads to prolonged “ringing” in the transducer after an excitation has been applied, which would be seen as noise in the measurement. It is commonly determined by two different methods, which often tend to give different results. One involves the minimum impedance |*Z*|_min_ at resonance, the free capacitance *C* (conventionally measured at 1 kHz) and the resonance and antiresonance frequencies [18]:

(3) $$Q_{m,Z}=\frac{f_{\text{a}}^{2}}{2\pi f_{\text{r}}\left|Z_{m}\right|C\left(f_{\text{a}}^{2}-f_{\text{r}}^{2}\right)}$$

This method is rather convenient, since all parameters except *C* can be determined from the impedance spectrum commonly used to determine the coupling coefficient. However, the value of |*Z*|_min_ is rather sensitive to spurious modes.

The second one is directly based on the 6 dB peak width as seen from the conductance spectrum, where *f*_1_ and *f*_2_ can be found as the maximum and minimum, respectively, of the susceptance close to the resonance [19]:

(4) $$Q_{\text{m},∆f}=\frac{f_{\text{r}}}{f_{2}-f_{1}}$$

Though not being insensitive to spurious modes, this method has the advantage that the operator can clearly see if a spurious mode is disrupting the measurement.

Finally, the acoustic impedance *Z*_a_ is found as the product of density and sound velocity from the thickness mode:

(5) $$Z_{\text{a}}=v\rho =2f_{\text{a},\text{t}}\rho$$

The common unit for *Z*_a_ is the megarayleigh, with 1 MRayl = 10^6^ kg/(m^2^·s).

Table 1 compares these properties of Pz37 before and after oil saturation to the two material types most commonly used for pulse-echo transducers: PZT 5A and K81 lead metaniobate. 

**Table 1:** Properties of Pz37 piezoceramic discs measured before and after oil impregnation. For all values stated, the relative standard deviation was less than 1.5%, except for *Q*_m_ for which it varied between 2.3% and 6.1%. Typical values for PZT 5A (commercial soft-doped PZT) and K81 (modified lead metaniobate from Meggitt Indiana) are given for comparison.

Category	Parameter	Unit	Pz37 Before Oil	Pz37 After Oil	PZT 5A Typical	K81 Typical
General	***ρ***	Mg/m^3^	6.02	6.22	7.70	6.00
	***Z*_a_**	MRayl	18.7	19.3	34	19
	***ε*_33,r_**	-	1058	1053	1700	300
	***T*_C_**	°C	365	365	365	460
Thickness Mode	***d*_33_**	pC/N	437	431	380	90
	***g*_33_**	mV·m/N	46.6	46.2	25.2	34.0
	***N*_t_**	km/s	1.28	1.34	2.0	1.5
	***Q*_m,*Z*_**	-	23	27	70	15
	***Q*_m,Δ*f*_**	-	90	97	200	-
	***k*_t_**	-	0.546	0.543	0.50	0.30
Planar Mode	***N*_p_**	km/s	1.67	1.66	2.0	2.0
	***Q*_m,*Z*_**	-	87	83	90	15
	***k*_p_**	-	0.357	0.368	0.60	0.07
Combined	***k*_p_/*k*_t_**	%	58	68	120	23

There are a number of points to note from these measurements. Starting with the density of Pz37, the density before oil infiltration indicates a porosity close to 25% (taking the theoretical density of PZT to be approximately 8 Mg/m^3^). Upon oil infiltration the density increases by 3.3%, which indicates a considerable degree of open porosity. This agrees well with the observation that in this type of system, the connectivity changes from 3-0 to 3-3 in the porosity range from 20% to 30% [10]. 

The measured value of the relative permittivity of Pz37, 1058, is intermediate between that of the standard soft PZT and K81. In order to understand the rather large decrease in permittivity, a comparison is useful with the work of Wersing *et al*. [3], who considered porous piezoceramics with a wide range of porosity and 3-3 or 3-0 connectivity. It was found that the set of formulae of Bruggeman for permittivity as a function of porosity describe these cases quite well and that the two types of connectivity differ by less than 6% up to a porosity of 30%. Specifically for piezoceramics with 3-3 connectivity and porosity *p* up to 60%, the following approximation is valid for the relative permittivity:

(6) $$\varepsilon \left(p\right)=\varepsilon \left(0\right)\left(1-\frac{3p}{2}\right)$$

where *ε*(0) is the relative permittivity of the dense piezoceramic. Taking the latter value to be 1700 and the porosity to be 25%, yields an expected relative permittivity of 1063, which is in good agreement with the measured value. 

Turning now to the piezoelectric properties, it is worth mentioning that thanks to a high *d*_33_ of the porous ceramics and the aforementioned reduced permittivity, Equation (1) yields a very high *g*_33_ coefficient compared to the other two types listed in Table 1. This is important for sensor applications using a voltage amplifier. 

The sensitivity at resonance relevant for the pulse-echo application is more adequately described by *k*_t_, and this is higher for Pz37 than for the dense soft PZT. It may appear counterintuitive that introducing an inactive phase could cause the thickness coupling coefficient to increase, but this has been observed earlier in comparisons with equivalent low-porosity discs [10,11] and confirmed by KLM-type modelling of composites of soft PZT and air, with connectivity ranging between 3-3 and 3-0. *k*_t_ generally showed a non-monotonous dependence on porosity and for both types of connectivity, a significant increase was seen in a wide range of porosity [20]. The impedance spectrum close to the thickness resonance showed an interesting variation with oil infiltration. To begin with, before oil infiltration these particular discs (with an aspect ratio of 0.1) showed spurious modes at the thickness resonance. Two minima in the absolute impedance, of almost equal depth, were seen, the one at lower frequency yielding a surprisingly high *k*_t_ ≈ 0.61 and the other one yielding an average *k*_t_ of 0.546. On the other hand, after oil infiltration, the first minimum (equivalent to *k*_t_ ≈ 0.61) was significantly less pronounced and a much cleaner resonance appeared yielding an average *k*_t_ of 0.543. Our interpretation is that the true *k*_t_ of the porous, dry material is close to 0.55 and that the introduction of oil might have damped the spurious mode. The observations reported here agree well with the findings in the interesting work on filling pores of piezoceramics with oil or araldite [21].

The planar coupling factor *k*_p_ shows a rather different dependence on porosity, namely a significant reduction. This is good agreement with previous observations and correlates with tendencies seen for transverse modes (*k*_31_, *d*_31_) [10]. For the pulse-echo application, planar coupling is undesired because harmonics of this may affect the thickness resonance, and indeed the very low *k*_p_ of K81 is considered an important virtue in this case. The measured value of 0.357 for Pz37 is between that of K81 and the standard soft PZT, and even after oil infiltration the *k*_p_/*k*_t_ ratio remains acceptable.

The most important point to note about the mechanical quality factors is that they are lower for Pz37 than for the standard soft PZT, at least for the thickness mode, and apparently exhibit a minor increase upon oil infiltration (although the *Q*_m_ measurement showed some scatter the tendency was consistent for all samples). The frequency constant shows a larger increase with porosity for the thickness mode than for the planar one [3], and as expected, the porosity dependence is even stronger for the acoustic impedance (*cf*. Equation (5)). *Z*_a_ of Pz37 is only slightly affected by oil infiltration and the values are very close to that of K81. The low acoustic impedance makes it easier to transfer acoustic energy to media such as water (*Z*_a_ 1.5 MRayl) and oil.

In summary the porous piezoceramic Pz37 offers significant improvements over standard soft PZT in a number of respects and it is clearly relevant to test the performance in a pulse-echo transducer.

#### 2.1.2. Transducer Characterisation

**Figure 2:**
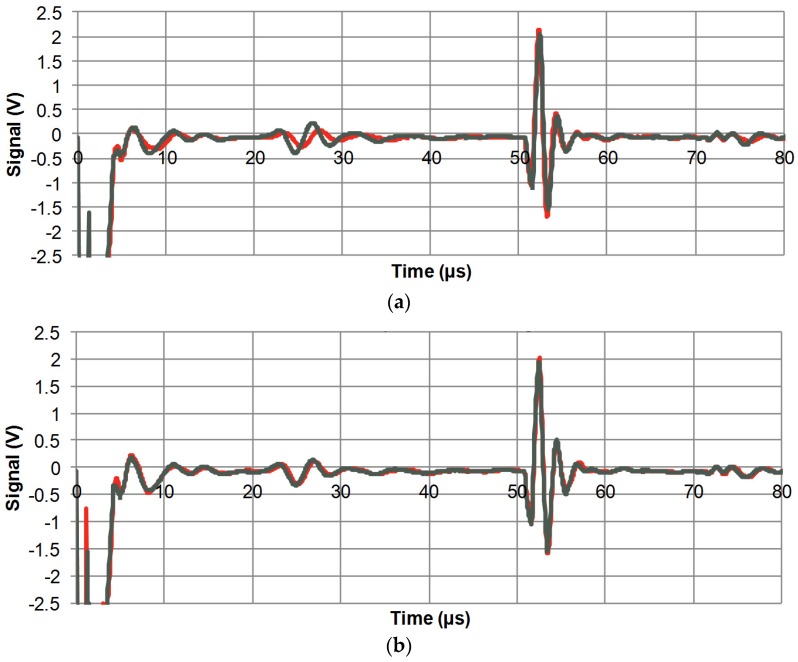
Pulse-echo response for bulk transducers (pulse voltage 100 V, gain −10 dB). (**a**) Two dry piezoceramic elements; (**b**) Two oil-saturated transducers.

With the porous bulk discs described in the previous section, 5 transducers of each type were manufactured as described in the experimental section. There was one clear, strong thickness mode at the nominal frequency, indicating good acoustic matching (*cf*. discussion of spurious modes above). The pulse-echo response of two such transducers built with dry piezo-elements is shown in Figure 2a. To begin with, some features of the curves should be pointed out. The strong negative signal (off-scale) at *t* < 5 µs is the excitation pulse, and between 5 and 10 µs the so-called main-bang ringdown is seen. This is the period where high acoustic energy reverberates in the piezo-element. The next feature, from 23 µs and slowly decaying for some 10 µs, is an echo coming from the backing material. A secondary backing echo starts at around 72 µs. The distinct peaks starting at around 51 µs are the target echo, which is of course the desired signal. The fact that the red and the grey curves show good agreement is a sign of reproducibility, and it should also be noted that the target echo is much stronger than the main-bang ringdown and the backing echoes. Figure 2b shows the pulse-echo response of two transducers with oil-saturated piezo-elements and in this case the two curves are nearly indistinguishable. Apart from this, a slightly higher amplitude in the main-bang ringdown can be seen. This could be associated with the apparent minor increase in the mechanical quality factors upon oil infiltration.

**Figure 3:**
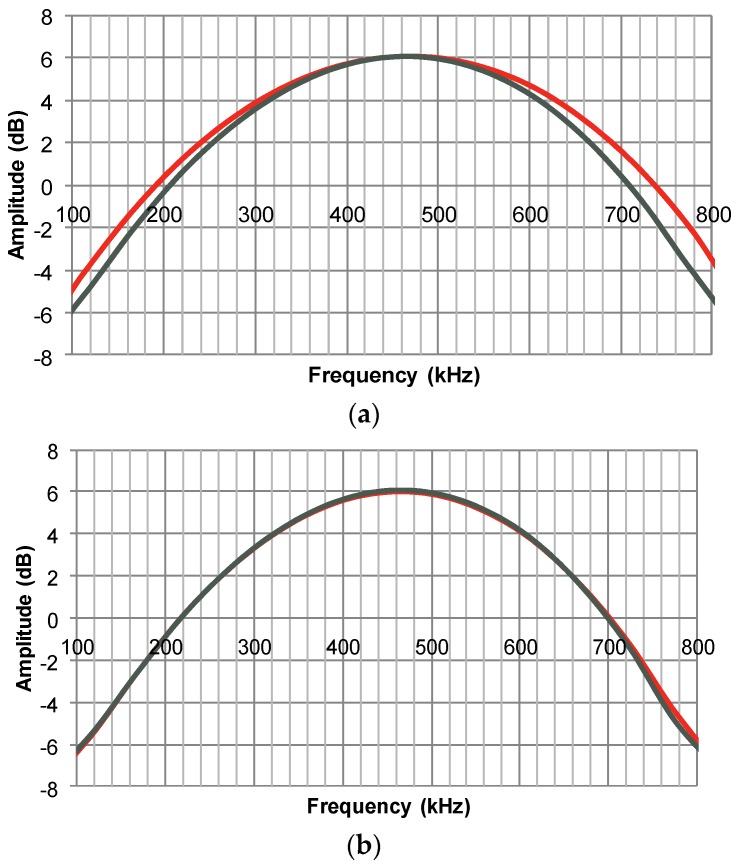
Normalised Fourier transforms of pulse-echo response of transducers. The −6 dB bandwidths can be found from the intersections with the 0 dB line. (**a**) Two dry piezoceramic elements; (**b**) Two oil-saturated transducers.

In order to examine the bandwidth of the pulses in Figure 2, a Fourier analysis is performed, and the result is shown in Figure 3. The fractional bandwidth (*i.e.*, the bandwidth found from the −6 dB points with respect to maximum amplitude, divided by the centre frequency) is above 100% for all four measurements, which is a very respectable performance for this application. A closer look reveals that the average of two similar transducers decreases from 114% to 105% as a result of the oil infiltration, and this is in good agreement with the slight decrease in *k*_t_ noted in the previous section.

In summary, the results obtained with the oil-saturated transducers based on the porous Pz37 are very promising for pulse-echo applications at elevated pressure, such as downhole drilling.

### 2.2. Thick-Film Transducers for Ultrasonic Imaging

Since thick films are closely integrated with a substrate, the characterisation of functional properties of the material itself is significantly more complicated than in the case of separate bulk elements. Therefore, this section will deal with functional characterisation of thick-film transducers only, and as will be seen, the main difference in terms of pulse-echo measurements is the higher frequencies involved. For some typical properties of the InSensor™ thick film used, the reader is referred to [22].

**Figure 4:**
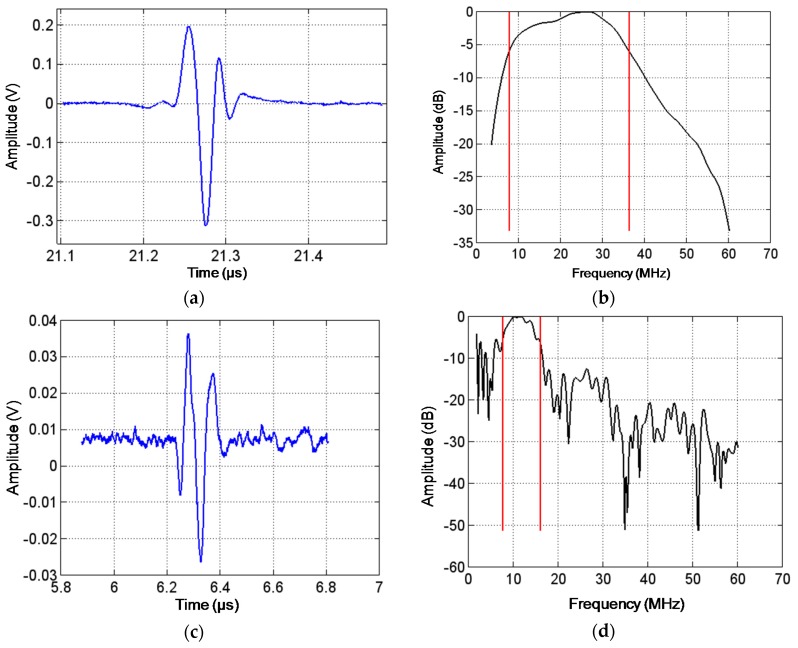
(**a**) Pulse-echo response of single-element thick-film transducer after excitation with a pulse with nominal amplitude of −300 V and duration of 5 ns; (**b**) Normalised Fourier transform of the signal shown in (**a**); (**c**) Pulse-echo response of one thick-film array element after excitation with a pulse with nominal amplitude of −300 V and duration of 30 ns; (**d**) Normalised Fourier transform of the signal shown in (**c**).

Figure 4a is a close-up view of the target echo from a pulse-echo measurement (with reflector placed in focal point) performed on a single-element thick-film transducer for ultrasonic imaging such as the one described in Section 3.2 below. The echo is seen to be very well-defined, and the Fourier analysis shown in Figure 4b reveals a −6 dB fractional bandwidth of 130% and a centre frequency of 22.1 MHz. This is a very good performance for high-resolution applications such as ultrasonic skin imaging. For this transducer the porosity plays a similar role as described in the section on porous bulk discs: increasing *k*_t_ for improved sensitivity; reducing the lateral coupling factor *k*_31_; reducing *Q*_m_ and thus improving the bandwidth; and reducing the acoustic impedance. It should be mentioned that for ultrasonic imaging, high bandwidth combined with high frequency translates into high resolution [15]. Because of the thick film being integrated with the substrate as mentioned above, there are important constraints on the substrate. To begin with, it should be chemically and physically compatible with the thick film at the processing temperatures, and furthermore it should play the role of a backing material, being well-matched acoustically to the film and able to dissipate acoustic energy. For the thick films described in this work, a porous ceramic substrate has been used.

In order to benefit from the many advantages offered by electronic beamforming, the logical next step is to move from single-element transducers to multi-element array transducers. Since thick films are generally deposited by high-resolution additive processes such as screen printing and pad printing, they offer great possibilities of high-level integration and cost-effective manufacturing. The thick-film array prepared in this work is an example of such a transducer with 32 elements defined by parallel lines of the top electrode. It is important to note that this is a so-called kerfless design, *i.e.*, the individual elements are not physically separated at the level of the thick film [23], which makes the patterning simpler and reduces the obtainable pitch (*i.e.*, centre distance between elements).

Pulse-echo measurements of the individual elements of 6 such arrays yield a centre frequency of 11.4 MHz and a −6 dB fractional bandwidth of 55% in average (see example in Figure 4c,d, with reflector 5 mm away), which is somewhat reduced in comparison with that shown in Figure 4a,b for a single-element transducer. The printing method is not quite the same for the flat 32-element array transducer as for the curved single-element transducer—the former is deposited by screen printing and the latter by pad printing—but experience shows that the two methods yield similar performance. Instead the reason should be sought in the less favourable geometry of the long, narrow line elements, and the lower target frequency that dictates a higher film thickness (performance is seen to decrease slightly for films with centre frequencies approaching 10 MHz). It is worth mentioning, however, that the fractional bandwidth of 55% compares well with the more conventional 2-2 composite transducers made by dicing bulk ceramic and backfilling with polymer (see for example [24]).

**Figure 5:**
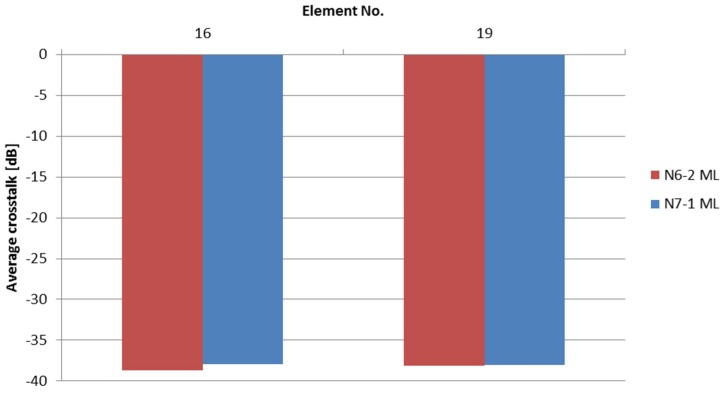
Cross-talk measured at time zero for two typical elements (No. 16 and 19) of two linear arrays. The average crosstalk is found to be approximately −38 dB.

For such a multi-element transducer, cross-talk is an important parameter to consider, especially in view of the kerfless design. This has been measured by comparing the voltage amplitude for the four elements closest to the exited element and an average value of −38 dB has been obtained. Figure 5 shows a comparison for two representative arrays. This is a surprisingly low cross-talk for a kerfless design, especially when compared with the value of −24 dB obtained with the 2-2 composite transducer mentioned before [24]. Clearly, the strongly reduced lateral coupling induced by porosity (as discussed in the case of *k*_p_ of the porous bulk discs) is crucial for obtaining such a low cross-talk with this kerfless design.

The successful use of these thick-film multi-element transducers for ultrasonic imaging is described in a recent paper [16].

## 3. Experimental Section

### 3.1. Bulk Transducers

A number of discs (diameter 24.6 mm, thickness 2.5 mm) were prepared from a commercial soft PZT, Ferroperm™ Pz37 (Meggitt Sensing Systems, Kvistgaard, Denmark) [17], which in this article will be referred to as Pz37. This ceramic type has been designed to have a high level of porosity (by means of an organic pore former), approximately 25%, in order to obtain specific acoustic properties. The discs were poled using the same poling field as used for an equivalent low-porosity soft PZT, which was well below the breakdown field of the porous material (thus no dielectric breakdown was observed). Some of the discs were infiltrated with silicone oil by using a pressure cycle: vacuum—overpressure—vacuum. The number of samples for each category (without or with oil impregnation) was 5.

**Figure 6:**
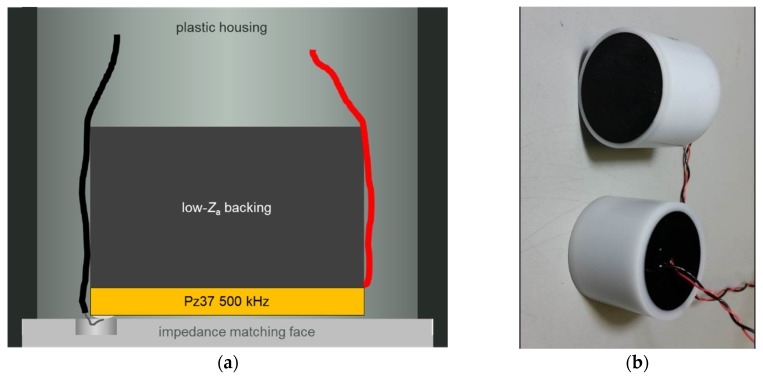
(**a**) Sketch of transducer design; (**b**) Photo of two assembled transducers.

For the transducer fabrication, wires were soldered to the disc before bonding it to a cylinder of backing material with similar diameter and embedding in a plastic housing as indicated in Figure 6a. In order to reduce back wall echoes, the impedance of the backing was matched to that of the ceramic. Finally, the front face was coated with an epoxy layer with a thickness corresponding to one quarter of the wavelength (for impedance matching), and the back side and electrical leads were protected by potting (Figure 6b). Again, for each category the number of transducers was 5.

For the characterisation of the discs, the following equipment was used: Hewlett-Packard 4278A capacitance bridge (Keysight Technologies, Santa Rosa, CA, USA), Agilent E4990A impedance analyser (Keysight Technologies, Santa Rosa, CA, USA), PiezoMeter System PM200 & PM300 *d*_33_ meter (Piezotest Ltd., London, UK).

**Figure 7:**
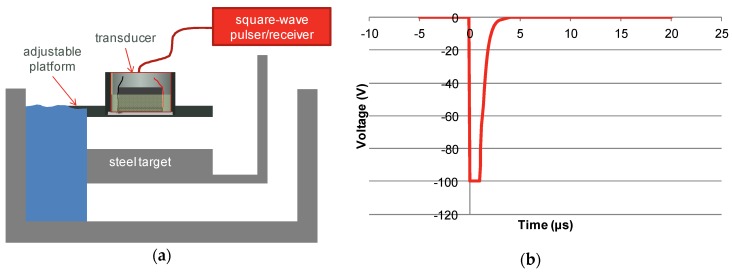
(**a**) Measurement set-up for pulse-echo measurement; (**b**) Drive pulse used.

The set-up for pulse-echo measurements is shown in Figure 7a. The platform supporting the transducer had adjustable angle with respect to the target surface. The signal was generated with a Panametrics 5077 square-wave pulser/receiver (GE Measurement, Boston, MA, USA). For most of the testing described in this paper, the signal was set at 100 V and the period of the pulse was varied to maximise the return signal (see Figure 7b).

### 3.2. Thick Films

**Figure 8:**
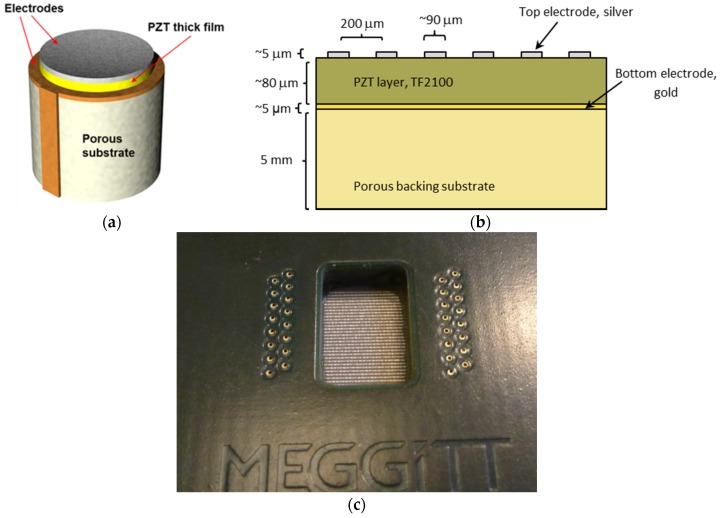
(**a**) Typical structure of a commercial high-frequency single-element transducer based on pad-printed piezoceramic thick film. The thickness of the piezoceramic film is approximately 20 µm; (**b**) Schematic drawing of structure of the linear array with a pitch of 200 µm, based on piezoceramic thick film; (**c**) Photo of the 32-element screen-printed linear array mounted on a printed circuit board. The overlapping length of the line elements is 5 mm. (**b**) and (**c**) © 2015 IEEE. Reprinted, with permission, from DOI 10.1109/ULTSYM.2015.0277.

The single-element thick-film transducer (Figure 8a) was manufactured by pad-printing a gold bottom electrode, InSensor™ TF2100 PZT thick film (Meggitt Sensing Systems, Kvistgaard, Denmark) [22] (approx. 20 µm thick) and silver top electrode onto a porous ceramic substrate. The multi-element array transducers shown in Figure 8b,c were prepared in a similar manner, except that screen printing was used and the thickness was approximately 90 µm. 6 arrays were manufactured, all showing similar performance.

The pulse-echo measurements were performed using a JSR Ultrasonics DPR500 dual pulser/receiver (Imaginant Inc., Pittsford, NY, USA) with remote pulsers in combination with an Agilent Infiniium DSO8064A oscilloscope (Keysight Technologies, Santa Rosa, CA, USA).

## 4. Conclusions

This work has shown two very promising and commercially relevant examples of the use of porous piezoceramics. It is clear that introducing and controlling porosity creates new functional materials that are able to compete with existing materials and also enable new devices.

For the thick films, it should be mentioned that pad printing and screen printing are additive and cost-effective manufacturing methods with very useful characteristics in terms of integration with a substrate also acting as a backing material.
